# Polyphenol supplementation boosts aerobic endurance in athletes: systematic review

**DOI:** 10.3389/fphys.2024.1369174

**Published:** 2024-04-08

**Authors:** Gexin Cao, Jing Zuo, Baile Wu, Ying Wu

**Affiliations:** ^1^ Department of Exercise Physiology, School of Sports Science, Beijing Sports University, Beijing, China; ^2^ Laboratory of Sports Stress and Adaptation of General Administration of Sport, Beijing Sports University, Beijing, China; ^3^ Department of Anatomy Laboratory, School of Sports Science, Beijing Sports University, Beijing, China

**Keywords:** polyphenols, athletic performance, aerobic endurance exercise, oxidative stress, systematic review

## Abstract

In recent years, an increasing trend has been observed in the consumption of specific polyphenols, such as flavonoids and phenolic acids, derived from green tea, berries, and other similar sources. These compounds are believed to alleviate oxidative stress and inflammation resulting from exercise, potentially enhancing athletic performance. This systematic review critically examines the role of polyphenol supplementation in improving aerobic endurance among athletes and individuals with regular exercise habits. The review involved a thorough search of major literature databases, including PubMed, Web of Science, SCOPUS, SPORTDiscus, and Embase, covering re-search up to the year 2023. Out of 491 initially identified articles, 11 met the strict inclusion criteria for this review. These studies specifically focused on the incorporation of polyphenols or polyphenol-containing complexes in their experimental design, assessing their impact on aerobic endurance. The methodology adhered to the Preferred Reporting Items for Systematic Reviews and Meta-Analyses (PRISMA) guidelines, and the risk of bias was evaluated using the Cochrane bias risk assessment tool. While this review suggests that polyphenol supplementation might enhance certain aspects of aerobic endurance and promote fat oxidation, it is important to interpret these findings with caution, considering the limited number of studies available.

**Systematic Review Registration:** https://www.crd.york.ac.uk/PROSPERO/, identifier CRD42023453321.

## 1 Introduction

Prolonged periods of high-intensity endurance training and competition can lead to exercise-induced fatigue in athletes (Sánchez Díaz et al., 2022), a decline in muscle function (Jun-Qing, 2018), and the initiation of oxidative stress (Morgan et al., 2019; Sánchez Díaz et al., 2022). Ultimately, these factors may impact athletic performance and activity levels. In recent years, professional sports teams and amateur enthusiasts have widely embraced natural plant extracts and phytochemicals to enhance their athletic performance, speed up post-exercise recovery, and maintain their overall physical health (Striegel et al., 2005; Kim et al., 2011; Darvishi et al., 2013; Knapik et al., 2016). Polyphenols represent a crucial category of natural botanical extracts, and there is mounting evidence to suggest their significant potential in enhancing athletic performance and aiding recovery (Bai et al., 2022; Liu et al., 2022; López-Torres et al., 2022; Sánchez Díaz et al., 2022; Roberts et al., 2023; Zare et al., 2023).

Polyphenols are micronutrients present in plants and their derivatives, such as berries, wine, green tea, and chocolate (Manach et al., 2004). In addition, they serve as secondary metabolites in plants that are involved in several critical processes such as growth, pigmentation, pollination, and defense against pathogens and environmental changes (Duthie et al., 2003). Due to their multifunctional effects on various physiological conditions in organisms, including oxidative stress (Ristow, 2014), chronic diseases (Mandel and Youdim, 2004; Lagouge, 2006), and immunity (Somerville et al., 2016), polyphenols have emerged as a rapidly growing area of research (Manach et al., 2004). Polyphenols consist of thousands of compounds, mainly identified by one or more hydroxy groups attached to one or more benzene rings. According to the number of phenolic rings and the connecting structural elements, four primary categories can be discerned, as indicated by reference (Bowtell and Kelly, 2019): phenolic acids, lignans, stilbenes, and flavonoids (Manach et al., 2004). The most common polyphenolic compounds from different categories and their main food sources are summarized in Table 1.

**TABLE 1:** Food sources of the different polyphenol categories and compounds.

Polyphenol categories	Compounds	Food source
Phenolic acids	Benzoic	Gallic acid–tea
Lignans	Enterodiol	Seeds, legumes
Stilbenes	Resveratrol	Grapes
Flavonoids	Epicatechin, Catechins	Cocoa, Green tea
	Quercetin, Gallotannins and more	Apples
		Mango and more

In recent years, polyphenols have been frequently associated with sports and exercise due to their antioxidant properties (Bojarczuk and Dzitkowska-Zabielska, 2022). The ability of polyphenols to scavenge free radicals is related to their chemical structure (Bowtell and Kelly, 2019). Phenolic hydroxyl groups can provide an electron to free radicals, while the aromatic rings in polyphenols can stabilize the resulting phenoxyl radicals (Bors et al., 2001). Polyphenols are metal chelators, which means they can reduce the formation of free radicals catalyzed by metals (Isabelle et al., 1994). However, their concentrations in the human body are relatively low, and plasma phenolic compounds appear to be unlikely to function as effective direct antioxidants within the body (Bowtell and Kelly, 2019). There is increasing evidence to suggest that the antioxidant properties of polyphenols are linked with the improvement of endogenous antioxidant capacity activated via the nuclear factor erythroid 2-related factor 2 (Nrf2) signaling pathway (Somerville et al., 2017; Bowtell and Kelly, 2019; Kitaoka, 2021; Bojarczuk and Dzitkowska-Zabielska, 2022). Nrf2 belongs to the Cap-N-Collar transcription factor family and has a substantial function in mitochondrial biogenesis. Nrf2 gene variants associated with endurance performance were also identified (Stevenson, 2012; Kitaoka, 2021). Under static/steady-state conditions, Nrf2 is continuously degraded through the ubiquitin-proteasome pathway mediated by Kelch-like ECH-associated protein 1 (Keap1) (Kitaoka, 2021). However, under stress conditions, Nrf2 translocates into the cell nucleus and binds to the antioxidant response elements (AREs) of target cell-protective genes. Studies utilizing Nrf2-deficient mice on a C57BL/6 background suggest the significance of Nrf2 for antioxidant enzymes in skeletal muscle (Corey et al., 2012; Narasimhan et al., 2013; Tryon et al., 2016; Ahn et al., 2018; Kitaoka et al., 2019). There is evidence suggesting that long-term consumption of polyphenols can increase the endogenous antioxidant system’s capacity through the Nrf2 signaling pathway and AREs pathway like the way exercise adaptation enhances the capacity of the endogenous antioxidant system (Bowtell and Kelly, 2019).

The addition of polyphenols has the potential to augment the body’s antioxidant capacity. For instance, male amateur runners, when consuming grape juice at a dose of 10 mL/kg/day for 2 h before exercising at 80% VO_2max_ intensity until fatigue, showed an increase in total antioxidant capacity (de Lima Tavares Toscano et al., 2020). Acute supplementation of 900 mg of cocoa flavanols also increased the total antioxidant capacity in well-trained male cyclists (Decroix et al., 2017). In addition, endurance exercise can cause oxidative damage that may restrict the vasodilation capacity of blood vessels and result in changes to blood rheology (Tofas et al., 2019). The ergogenic effects of polyphenols seem to be related to alterations in vascular function. For instance, previous research has highlighted the beneficial effects of pre-supplementation with pomegranate juice on brachial artery blood flow and vascular diameter before testing (Roelofs et al., 2017). Similarly, in a long-term study lasting 4 weeks, daily supplementation of a total of 571 mg of green tea extract rich in epigallocatechin gallate (EGCG) resulted in a certain degree of increase in VO_2max_, while maximum cardiac output remained unaffected by EGCG (Roberts et al., 2015). This suggests that the enhancement in exercise performance could be attributed to an increase in the blood oxygen difference between arteries and veins. In the muscles engaged during exercise, there was a significant improvement in oxygen transport efficiency, greatly enhancing muscle perfusion capacity (Bowtell and Kelly, 2019). Nitric oxide (NO) functions as a cellular messenger both intracellularly and extracellularly, and recent studies suggest that polyphenols regulate specific cellular mechanisms by promoting endothelial NO synthesis, which in turn leads to vasodilation and increased blood flow (Maiorana et al., 2003; Webb et al., 2008). For these noticeable vascular effects, the most plausible mechanism is likely to either reducing ROS generation or enhance ROS detoxification capability through the antioxidant system. As the reaction between superoxide and NO diminishes the production of peroxynitrite, the reduction in ROS exposure would enhance the bioavailability of the effective vasodilator NO. Therefore, in certain sports disciplines, supplementing with polyphenols to improve endothelial function and vasodilation, hence enhancing hemodynamics, could potentially improve athletic performance (Labonté et al., 2013; Bowtell and Kelly, 2019).

Reactive oxygen species (ROS) are generated in skeletal muscles during both rest and exercise and serve as ubiquitous cellular signals within the mitochondria of all mammalian cells (Maraldi, 2013; Kitaoka, 2021). Under normal circumstances, these ROS are buffered by the cell’s internal antioxidant systems to prevent the accumulation of oxidative damage. Recently, there has been some debate regarding exercise-induced oxidative stress, stemming from the dual role of reactive oxygen species in cellular biology (Powers et al., 2020). On one hand, excessive levels of ROS are associated with oxidative stress, leading to cell damage, aging, and various diseases (Kruk et al., 2019). On the other hand, moderate levels of ROS are essential for regulating important physiological processes, including stress responses (such as exercise) that promote synthesis and metabolic adaptations (Radak et al., 2005; Musci et al., 2019). The concept of hormesis explains how low to moderate levels of ROS can promote cellular adaptive responses, improving functionality (Radak et al., 2005). Hormesis refers to a biphasic dose-response relationship, where low doses of a potentially harmful factor (like ROS) stimulate beneficial adaptive responses, enhancing cell function and survival, while high doses are detrimental. This concept suggests that exercise-induced ROS production, below a certain threshold, is necessary for triggering cellular adaptations leading to performance enhancement, muscle growth, and increased metabolic efficiency (Calabrese and Baldwin, 1999; Mattson, 2008). Exercise-induced ROS play a key role in muscle adaptation, promoting mitochondrial biogenesis and antioxidant defenses (Powers et al., 2010). However, identifying the optimal levels of ROS that promote health rather than harm remains a challenge. Current understanding calls for a balanced approach, allowing for a certain degree of ROS production to stimulate beneficial synthetic and metabolic pathways, without causing oxidative damage. This balance is likely highly individualized, influenced by factors such as genetics, lifestyle, and the type and intensity of exercise. During repetitive muscle contractions, ROS are continuously generated in an intensity-dependent manner by various enzymatic sources, such as NADPH oxidase (NOX) (Reid, 2016). NOX is currently the only known enzyme family with the sole function of ROS production (Maraldi, 2013), and it plays a crucial role in generating superoxide, one of the key sources of oxidative stress. Polyphenols can reduce the formation of peroxynitrite by inhibiting NOX (Maraldi, 2013). Consequently, this enhances endogenous antioxidant capacity and preserves the bioavailability of nitric oxide (NO) (Zare et al., 2023). Therefore, the ergogenic effects of polyphenols seem to be supported by vascular and antioxidative mechanisms.

Due to the benefits and characteristics of polyphenols mentioned above, research on polyphenols in the field of sports is becoming increasingly popular. Although there is currently a substantial body of research, most reviews tend to focus on recovery from exercise-induced muscle damage (EIMD) (Martin-Rincon et al., 2020; Carey et al., 2021; Huang et al., 2021; Ortega et al., 2021; Sánchez Díaz et al., 2022) or the effects of specific types of polyphenols on exercise (Braakhuis et al., 2020; Corr et al., 2021). Reviews addressing effects on exercise performance lack categorization and discussion of effects on different types of exercise, and effects on endurance or explosive exercise performance remain unclear (Vafaee et al., 2019; Braakhuis et al., 2020; D'Unienville et al., 2021; Bojarczuk and Dzitkowska-Zabielska, 2022; Sánchez Díaz et al., 2022). The intake of exogenous polyphenols may upregulate the endogenous antioxidant defense system, but the effects of different supplementation methods and dosages require further discussion. This review summarizes current research on the effects of polyphenols or polyphenol compounds on endurance exercise performance and identifies their effects, providing a basis for their potential application in endurance activities (amateur or non-amateur).

## 2 Methods

This systematic review follows the Preferred Reporting Items for Systematic Reviews and Meta-Analyses 2020 (PRISMA 2020) guidelines (Page et al., 2021). The study has been assessed and registered in the International Prospective Register of Systematic Reviews (PROSPERO) under the registration number CRD42023453321, as of 30 August 2023.

### 2.1 Search strategy

The search for relevant studies was conducted in several databases, including PubMed, Scopus, Web of Science (WOS), EBSCO-SPORTDiscus, and Embase. The search covered the period from the inception of the databases to August 2023. The search strategy employed both subject headings and Boolean operators, primarily focusing on two main concepts: polyphenols and exercise performance. As an example, for the PubMed database, the search equation was as follows: (“Athletic Performance” [Mesh] OR Athletic Performances OR Performance, Athletic OR Performances, Athletic OR Sports Performance OR Performance, Sports OR Performances, Sports OR Sports Performances) AND (“Polyphenols” [Mesh] or Polyphenol or Provinols).

### 2.2 Eligibility criteria

The inclusion and exclusion criteria for this systematic review are well defined according to the PICOS model: Population (P): healthy athletes and sports enthusiasts Intervention; (I): Supplementation with polyphenols or polyphenol compounds, including combinations of several polyphenols, with clear supplementation doses; Comparison (C): Comparison between intervention/experimental groups with similar characteristics and a placebo group; Outcome (O): Studies that include tests of athletic or sports performance before and after supplementation with polyphenols or polyphenol compounds, with available test data; Study (S): Randomized controlled trials with a parallel or crossover design, published in English. In addition, exclusion criteria were specified as follows: (i) Studies with confounding factors other than polyphenols. (ii) Studies with types of exercise programs that were not consistent with the focus of the research. (iii) Supplements containing non-standardized polyphenol compounds or supplements with unknown polyphenol content. (iv) Studies with incomplete or missing subject information, experimental protocols, or procedures. (v) Studies with no data available for extraction. (vi) Inaccessible full-text articles by legitimate means. (vii) Studies conducted in specific environments not relevant to the research.

### 2.3 Study selection

The search for relevant published literature was conducted jointly by two authors in a standardized manner to assess the eligibility of the literature. Any disagreements that arose during the screening process were resolved through discussion and negotiation. During the screening process, all duplicate articles and non-clinical trial literature were removed. The screening process included the examination of titles and abstracts of the literature, including relevant articles that contributed to the research. Finally, the literature that met the inclusion criteria was selected for full-text reading and review.

### 2.4 Data extraction

After applying the eligibility criteria, the following information was extracted from each included literature: Bibliographic information (author(s) and year of publication), study design: (whether the study design was parallel or crossover, information on blinding of subjects, sample size), population characteristics (age of participants, gender distribution, height and weight data, if available), details of polyphenol supplementation: (types of polyphenol supplements used, dosage of polyphenol supplements, timing and method of supplementation). (i) Performance measures: time to complete the trial, time to fatigue during exercise, power output, distance covered, speed achieved, maximum aerobic velocity (MAV), Rate of Perceived Exertion (RPE), exercise economy, training intensity. (ii) Metabolic metrics: maximum oxygen consumption (VO2max), oxygen consumption (VO2), carbon dioxide production (VCO2), respiratory rate (RR), heart rate (HR), energy metabolism parameters such as carbohydrate oxidation (CHOox) and fat oxidation (FATox), blood glucose concentration (glucose), blood lactate concentration (lactate), blood lactate concentration at certain points (B [La]). (iii) Antioxidant Capacity Metrics: high-density lipoprotein (HDL) levels, low-density lipoprotein (LDL) levels, total antioxidant capacity (TAC), aspartate aminotransferase (AST) levels (U/L), and other relevant markers of antioxidant capacity.

## 3 Results

### 3.1 Study selection

**FIGURE 1:**
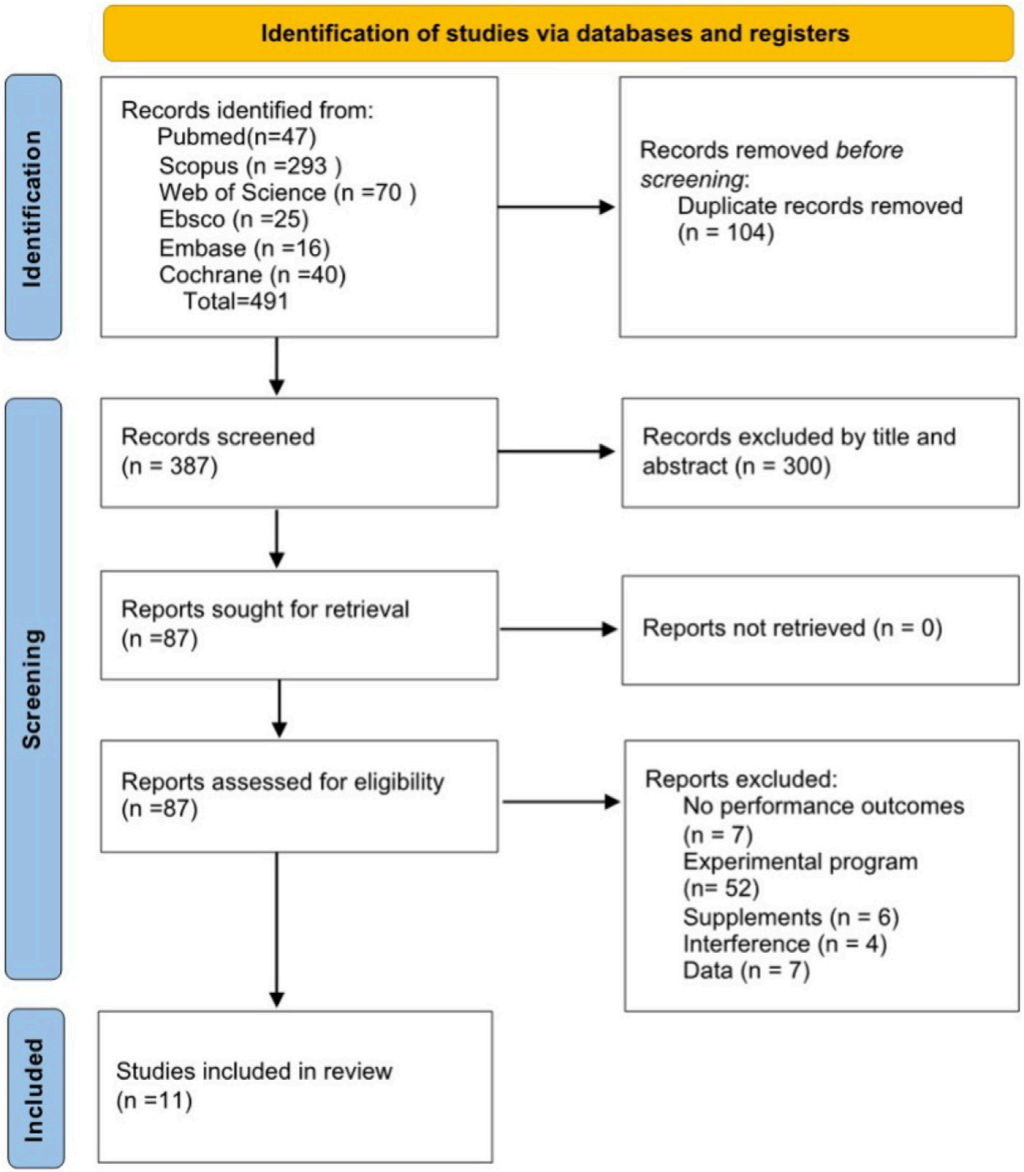
Selection of studies according to an adapted version of PRISMA 2020 flow diagram.

A total of 11 trials were included in this systematic review. Of these 11 trials, seven had male participants and three had mixed-gender groups. The initial search of Scopus, PubMed, Web of Science (WOS), SportDiscus, Embase, and Cochrane yielded 491 relevant articles. After removing 104 duplicate articles, 387 remained. After reviewing the titles and abstracts, 300 articles were excluded as they did not meet the inclusion criteria. A total of 87 studies were reviewed, of which 76 were subsequently excluded (for reasons outlined in the flowchart, Figure 1).

### 3.2 Characteristics of the studies

#### 3.2.1 Method

Of the 11 trials included in this review, five used a parallel design and six used a crossover design (Table 2). In the selected trials, participants were randomly assigned to either the placebo group or the experimental group. In nine of these trials, both researchers and participants were unaware of the treatment allocation, in one trial researchers were aware of the intervention allocation, and in one trial participants were aware that they were receiving the intervention.

**TABLE 2:** RCT design characteristics for all studies included in this review.

Author	Study design	Characteristics	Kind	Dose, timing	Polyphenolic content	Exercise	Physical performance test	Metabolic parameters	Anti-oxidation parameters
Nho and Kim. (2022)(Nho and Kim, 2022)	RCT double-blind crossover	I collegiate basketball players (n = 12) 20. 2 ± 1	GSE	300 mg per day for 14 days		two bouts of cycling exercise at constant submaximal workloads	submaximal exercise of 120% of VO_2peak_: time↑	submaximal exercise of50%, 80%, 120% of VO_2peak_: HR↔, VCO_2_↔, RR↔, Vt↔, VE↔. RER↔, FMD↑ submaximal exercise of80%, 120% of VO_2peak_: VO_2_↑	-
Roberts et al. (2015)(Roberts et al., 2015)	RCT double-blind, parallel	recreationally active males (n = 14), 21. 4 ± 0. 3	dGTE	571 mg per day for 4 weeks	400 mg/dEGCG, 91. 21% total catechins	submaximal assessment and performance stage	submaximal exercise: distance covered↑, average power output↑, RPE↓	submaximal exercise: VO_2_↔, VCO_2_↔, RER↓, HR↓, SBP↔, DBP↔, FAT_tot_↑, CHO_tot_↓, TFA concentration ↔, total fat acid concentration ↔	-
Cook et al. (2015)(Cook et al., 2015)	RCT double-blind crossover	healthy men with a history of sport participation of greater than 3 years (n = 14), 38 ± 13	NZBC	300 mg per day for 7 days	300 mg contains 105 mg anthocyanin	30 min cycling protocol, 16. 1 km best effort time-trial	Submaximal exercise at 45%VO_2max_, 55% VO_2max_, 65%VO_2max_: power↔, cycling economy ↔.16. 1 km cycling time-trial: completion time↓	Submaximal exercise at 45%VO2max, 55% VO_2max_, 65% VO_2max_	-
								VO_2_↔, VCO_2_↔, HR↔, RER↓, Lactose↔, glucose↔, EE↔, CHOox↔, FATox↑16. 1 km cycling time-trial: HR↔, cadence↔	
Deley et al. (2017)(Deley et al., 2017)	RCT double-blind, crossover	healthy physically active males (n = 48), 31. 0 ± 6. 0	Vinitrox™	2 capsules of 250 mg preceding evening and 1 hour before the endurance test	At least 300 mg/day	two endurance tests	endurance test: time to exhaustion↑, time to reach maximal perceived exertion↑	endurance test HR↔, VO_2_↔, VE↔, SBP_max_↔, DBP_max_	-
Gaamouri et al. (2019)(Gaamouri et al., 2019)	RCT double-blind parallel	taekwondo athletes (n = 23), 21. 9 ± 1. 2	carob pods	40 g per day for 6 weeks	40 g contains208 mg of total polyphenol, 14. 4 mg of flavonoids	yo-yo intermittent recovery test level-1	yo-yo intermittent recovery test: distance↑, MAV↑, RPE↑	yo-yo intermittent recovery test: HR↔	-
Martínez-Noguera et al. (2019)(Martinez-Noguera et al., 2019)	RCT single-blind crossover	healthy male amateur cyclists (n = 15), 33. 3 ± 7.9	Cardiose^®^	500 mg during and after a rectangular test	500 mg	Rectangular Test. Repeated Sprints Test	The best date of Repeated sprint test: PeakPower↔, Poweraverage↑, Time to peakpower↔, Max speed↑, total energy↑ the average date of Repeated sprint test: PeakPower↔, Poweraverage↔, Time to peakpower↔Max speed↔, total energy↔	VT1 VO2↔, VCO2↔, RER↔, HR↔, efficiency↔, carbohydrates↔	CAT↔, SOD↔, GSH↔, GSSG↔, % GSSG/GSH↔, TBARS↔
Willems et al. (2015)(Willems et al., 2015)	RCT single-blind crossover	trained triathletes with >3 yrs experience (n = 13) (male = 8, female = 5)38 ± 8	New Zealand blackcurrant	6 g per day for 7days	6 g: 138. 6 mg anthocyanin	Incremental Cycling Protocols	incremental cycling 1 mmol/L lactate rise: intensity↑, 4 mmol/L OBLA: intensity↑	incremental cycling: DPB↔, SBP↔, MAP↔, HR↔, SV↔, CO↔, Plasma lactate↓	-
de Lima Tavares Toscano et al. (2020)(de Lima Tavares Toscano et al., 2020)	RCT double-blind, crossover	recreational male runners (n = 14), 39 ± 9	grape juice	10 mL/kg/day for 7 days and 2 h before test	Total phenolics: 3,106. 6/mg/L	Run to exhaustion test	time to exhaustion↑ distance ↑	fat↔, cho↔	antioxidant activity ↑ lipid peroxidation response ↔
Howatson et al. (2022)(Howatson et al., 2022)	RCT double-blind parallel	male recreational runners (n = 30), 33 ± 7	Haskap berries	6 g per day for 6 days	anthocyanin content was ∼24. 9 mg/g	5 km treadmill TT, a submaximal lactate profile	submaximal test: speed↔, RPE↔5 KM time trail: mean speed↑, time↓, RPE↔	LT at submaximal test	-
								HR↓, absolute VO2↑, relative VO2↓	
								LTP at submaximal test	
								HR↓, absolute VO2↔, relative VO2↔	
								5 KM time trail: lactate↔	
Roberts et al. (2023)(Roberts et al., 2023)	RCT double-blind parallel	recreationally active participants (n = 29) 42 ± 2	OliP	56 mL per day for 16 days	28 mL contains 315. 9 mg Phenolic Profile	Submaximal and Performance Test	demanding aerobic session: exercise intensity ↔, Economy ↔	Aerobic session: VO_2_↔, VCO_2_↔, VE↔, RER↔, %of baseline VO_2max_↔, Kcal/d↔, CHO↔, FAT↔, PRO↔.onset of submaximal exercise: VO_2_↔	-
						Demanding Aerobic Session	Onset of submaximal exercise: τ↓	LT1of submaximal exercise: VO_2_↓,%oVO_2max_ of baseline↓, VCO_2_↔, VE↔, RER↔, B [La] ↔	
							LT1 of submaximal exercise: economy↓, RPE↔	LT2 of submaximal exercise%VO_2max↔_, VO_2_↔, VCO_2_↔, VE↔, RER↔, B [La] ↔	
							LT2 of submaximal exercise: economy↔, RPE↓		
Morgan et al. (2019)(Morgan et al., 2019)	RCT double-blind parallel	trained male cyclists (n = 8), 19. 7 ± 1. 6	MC	462. 8 mg per day for 7 days	462. 8mg contains 257 mg anthocyanin	10-min steady-state cycling at ∼ 65% VO2peak,15-km Time Trial on two occasions	15 km time-trial: completion time ↓	steady stage exercise:baseline TOI↑, RER↔, mean SS exercise TOI ↔, mean VO2↔, Lactate↑ 15 km time-trial: RER↔, TOI ↔, mean VO2↔, Lactate↔	-

- content not specified: ↔ no significant difference; ↑ significantly higher than placebo group; ↓ significantly lower than placebo group; abbreviations: RCT, randomised controlled trials; HR, heart rate; RR, respiratory rate; Vt, tidal volume; VE, minute ventilation; VO2max, maximal oxygen uptake; VO2, volume of oxygen; VCO2, volume of carbon dioxide; RER, respiratory exchange ratio; FMD, flow-mediated dilatation; CHOtot, total carbohydrate oxidation; FATtot, total fat oxidation; EE, energy expenditure; SBP, systolic blood pressure; DBP, diastolic blood pressure; TFA, total fatty acid; CHOox, carbohydrate oxidation; FATox, fat oxidation; τ, time constant; RPE: rate of perceived exertion; B [La], blood lactate concentration; CHO, carbohydrate; PRO, protein; TOI, tissue oxygenation index; MAV, maximal aerobic velocity; OBLA, onset of blood plasma lactate accumulation; MAP, mean arterial pressure; SV, stroke volume; CO, cardiac output; TPR, total peripheral resistance; CAT, catalase; SOD, superoxide dismutase; GSH, reduced glutathione; GSSG, glutathione peroxidase; % GSSG/GSH, % glutathione peroxide/reduced glutathione; TBARS, thiobarbituric acid reactive substances.

#### 3.2.2 Participants

A total of 220 participants were included in the study. Of these, 164 were recreational athletes and 56 were professional athletes. There was only one study with a mixed sample, including female participants (5 women and eight men), without a gender comparison analysis. The age range of the studies included in the review was approximately 18–48 years.

#### 3.2.3 Intervention

As previously mentioned, among the eleven studies, nine compared the placebo group with the experimental group, while two studies conducted comparisons before and after intervention. Polyphenol-rich concentrated substances such as grape seed extract (GSE) (Nho and Kim, 2022), decaffeinated green tea extract (dGTE) (Roberts et al., 2015), New Zealand blackcurrant (NZBC) (Cook et al., 2015; Willems et al., 2015), organic olive fruit water phytocomplex (OliP) (Roberts et al., 2023), Montmorency cherry powder (MC) (Morgan et al., 2019), carob pods (Gaamouri et al., 2019), Haskap berries (Howatson et al., 2022), Vinitrox™(a combination of specific profile polyphenols from grape and apple) (Deley et al., 2017), Cardiose^®^(Martinez-Noguera et al., 2019), grape juice (de Lima Tavares Toscano et al., 2020). These eleven trials used specific polyphenols and combinations of polyphenols, and the forms of intake varied, five trials of polyphenol supplementation were in capsule form (Cook et al., 2015; Roberts et al., 2015; Deley et al., 2017; Morgan et al., 2019; Nho and Kim, 2022), and three trials were in water (Willems et al., 2015; Gaamouri et al., 2019) or yogurt-soluble form (Howatson et al., 2022) as the intervention, while other forms of intake included snacks (de Lima Tavares Toscano et al., 2020) and canned products (Roberts et al., 2023).

### 3.3 Results

#### 3.3.1 Sports performance results

Of the eleven studies included in this systematic review, three measured test completion time (Cook et al., 2015; Morgan et al., 2019; Howatson et al., 2022), three assessed exercise to fatigue time (Deley et al., 2017; de Lima Tavares Toscano et al., 2020; Nho and Kim, 2022), and three assessed power output-related metrics (Cook et al., 2015; Roberts et al., 2015; Martinez-Noguera et al., 2019). One study reported a specific metric, τ (time constant) (Roberts et al., 2023). Three studies measured distance (Roberts et al., 2015; Gaamouri et al., 2019; de Lima Tavares Toscano et al., 2020). Two trials assessed speed (Martinez-Noguera et al., 2019; Howatson et al., 2022). Two studies measured exercise intensity (Willems et al., 2015; Roberts et al., 2023), two studies provided an indicator of exercise economy (Cook et al., 2015; Roberts et al., 2023), and four studies assessed RPE (Roberts et al., 2015; Gaamouri et al., 2019; Howatson et al., 2022; Roberts et al., 2023). Only one study used MAV to measure aerobic work capacity (Gaamouri et al., 2019).

#### 3.3.2 Metabolic parameters

Of these eleven studies, ten assessed cardiorespiratory function (Cook et al., 2015; Roberts et al., 2015; Deley et al., 2017; Gaamouri et al., 2019; Martinez-Noguera et al., 2019; Morgan et al., 2019; Howatson et al., 2022; Nho and Kim, 2022; Roberts et al., 2023), and five assessed energy substances and their oxidation (Cook et al., 2015; Roberts et al., 2015; Martinez-Noguera et al., 2019; de Lima Tavares Toscano et al., 2020; Roberts et al., 2023). Five assessed lactate levels (Cook et al., 2015; Willems et al., 2015; Morgan et al., 2019; Howatson et al., 2022; Roberts et al., 2023). Only one study evaluated TOI (Morgan et al., 2019).

#### 3.3.3 Antioxidant parameters

Two of the eleven studies assessed antioxidant parameters, one assessed CAT, SOD, GSH, GSSG, TBARS (Martinez-Noguera et al., 2019), and one assessed antioxidant activity, lipid peroxidation (de Lima Tavares Toscano et al., 2020).

### 3.4 Risk of bias

**FIGURE 2:**
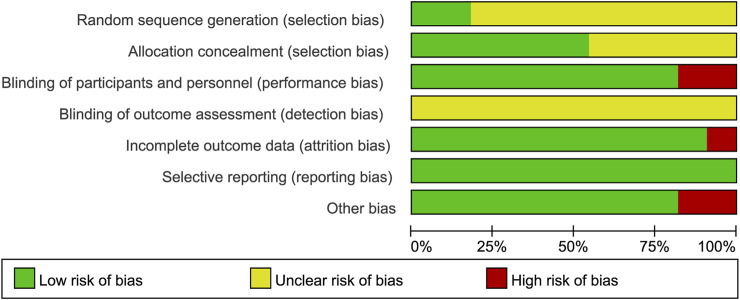
Risk of bias graph: Review of authors’ judgment on each risk of bias item from Cochrane Handbook for Systematic Reviewers (version 5. 1. 0) presented as percentages across all included studies.

**FIGURE 3:**
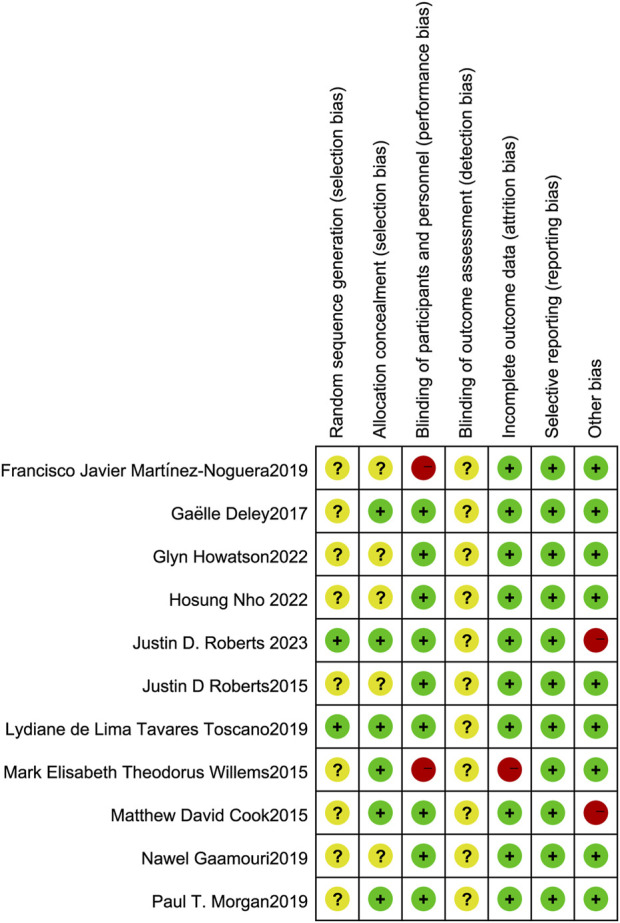
Risk of bias summary: Review of authors’ judgment on each risk of bias item from Cochrane Handbook for Systematic Reviewers (version 5. 1. 0).

The two authors used the Cochrane Collaboration tool from the Cochrane Handbook for Systematic Reviews of Interventions (version 5. 1. 0) to assess the risk of bias in the included randomized controlled trials (RCTs). The assessment criteria included: (i) Sequence generation (selection bias). (ii) Concealment of allocation (selection bias). (iii) Blinding of participants and staff (performance bias). (iv) Blinding of outcome assessors (detection bias). (v) Incomplete outcome data (attrition bias). (vi) Selective reporting (reporting bias). (vii) Other biases. Each criterion was rated at three levels: low risk, unclear risk, and high risk, to assess the risk of bias in each study (Figures 2, 3).

### 3.5 Synthesis of results

#### 3.5.1 Physical performance test

Table 2 shows that three trials assessed exercise time, and all reported a significant reduction in exercise completion time (Cook et al., 2015; Morgan et al., 2019; Howatson et al., 2022). When assessing aerobic endurance capacity using the time to fatigue indicator, three trials showed that the experimental groups had significantly longer fatigue times than the placebo groups (Deley et al., 2017; de Lima Tavares Toscano et al., 2020; Nho and Kim, 2022). Another time-related measure, time to peak power output, was measured in two trials, with one showing a significant increase (Deley et al., 2017), and the other showing no significant difference (Martinez-Noguera et al., 2019). Endurance running distance was assessed in three trials, two of which showed a significant positive effect of the intervention compared to placebo (Roberts et al., 2015; de Lima Tavares Toscano et al., 2020). In addition, one study showed a significant improvement compared to the pre-supplementation baseline (Gaamouri et al., 2019). Of the three trials that assessed power output indicators, one trial showed significantly higher results than the placebo group (Roberts et al., 2015), and one trial showed no significant difference (Cook et al., 2015). In the last of the three trials, there was no significant difference in peak power compared with the placebo group in the best date and the average date, despite significantly higher mean power in the experimental group of the best date, not the average date. This study also assessed total energy produced, with a significant increase in total energy produced during the best date of the repeated sprint compared to the placebo, although the overall average data during the competition showed no significant difference (Martinez-Noguera et al., 2019). Two studies assessed exercise intensity, with one study showing no significant difference compared to pre-supplementation (Roberts et al., 2023), and the other showed a significant improvement compared with the control group (Willems et al., 2015). In the two studies that evaluated the economy, one study showed no significant change compared with the placebo group (Cook et al., 2015). In another study, improvements were observed at the demanding aerobic session and the first lactate turning point compared with pre-supplementation levels, but there was no significant difference at the second lactate turning point (Roberts et al., 2023). Four studies used RPE as an effective measure of aerobic endurance capacity. The result of one paper was a significant decrease in RPE in the polyphenol supplement group compared to the control group (Roberts et al., 2015). One study showed no significant difference at the first lactate turning point compared to pre-supplementation levels, but a significant decrease at the second lactate turning point (Roberts et al., 2023). Another study also showed a significant increase compared to pre-supplementation (Gaamouri et al., 2019). In the last of these three studies, there was no significant change in RPE during exercise testing compared with the placebo group (Howatson et al., 2022). Speed is a valuable indicator of exercise performance. In one study, there was no significant difference in subjects’ speed during submaximal testing compared to placebo, but there was a significant improvement in the 5K time trial (Howatson et al., 2022). In another study using this speed indicator, the best performance for maximal speed was significantly improved compared to the placebo, but the average data for maximal speed showed no significant difference (Martinez-Noguera et al., 2019). One study used the time constant as an indicator and showed significant differences at the beginning of exercise after the intervention (Roberts et al., 2023). Only one study assessed MAV and showed a significant improvement compared with pre-supplementation (Gaamouri et al., 2019). In trials involving female athletes, no sex differences were analyzed for any of the physical performance variables (Deley et al., 2017).

#### 3.5.2 Metabolic parameters

When HR was assessed in eight trials, two of them showed a significant decrease compared with the placebo group (Roberts et al., 2015; Howatson et al., 2022). Of the remaining six trials, one did not show a significant difference compared with pre-supplementation values (Gaamouri et al., 2019). The results of the other trials showed no significant change compared with the placebo group (Cook et al., 2015; Willems et al., 2015; Deley et al., 2017; Martinez-Noguera et al., 2019; Nho and Kim, 2022). In addition, when VO_2_ was assessed in eight trials, one trial showed a significant increase compared with the placebo group (Nho and Kim, 2022). In the remaining five trials, there was no significant change in VO_2_ during the exercise test compared with placebo (Cook et al., 2015; Roberts et al., 2015; Deley et al., 2017; Martinez-Noguera et al., 2019; Morgan et al., 2019). In one study, VO_2_ did not show a significant difference compared to pre-supplementation during high-intensity aerobic exercise, submaximal exercise onset, and the LT2 of submaximal exercise. However, there was a significant decrease in VO_2_ at LT1 during submaximal exercise (Roberts et al., 2023). Another study showed a significant increase in absolute VO_2_ at the LT point of the submaximal test, a significant decrease in relative VO_2,_ and no significant change at the LTP point of the submaximal test. Relative VO_2_ has no significant difference during the whole exercise (Howatson et al., 2022). Of the five trials that evaluated VCO_2_, four showed no significant difference compared to placebo (Cook et al., 2015; Roberts et al., 2015; Martinez-Noguera et al., 2019; Nho and Kim, 2022), and only one trial showed no significant change in VCO_2_ over the entire study period compared to pre-supplementation (Roberts et al., 2023). When assessing RER measurements about VO_2_ and VCO_2_ in five trials, two trials showed a significant decrease compared to the placebo group (Cook et al., 2015; Roberts et al., 2015), while three trials showed no significant change compared to the placebo group (Martinez-Noguera et al., 2019; Morgan et al., 2019; Nho and Kim, 2022). Of note, one study showed no significant difference in RER compared with pre-supplementation (Roberts et al., 2023). Of the three studies that evaluated indicators of pulmonary ventilation, two found no significant difference in VE compared with the placebo group (Deley et al., 2017; Nho and Kim, 2022). One study also found no significant difference compared to before supplementation, and this study also assessed VE/VO_2_ and VE/VCO_2_ and found no significant differences (Roberts et al., 2023). Another measure related to RER was examined in one article, RR, which showed no significant difference compared to the control group (Nho and Kim, 2022). Three trials evaluated systolic and diastolic blood pressure as effective measures of aerobic performance and showed no significant differences compared to placebo (Roberts et al., 2015; Deley et al., 2017). Another study evaluated three indicators of cardiovascular function, SV, CO, and MAP except DPB and SBP, but found no significant differences compared with placebo (Willems et al., 2015). Lactate levels were assessed in five trials, two of which showed no significant difference compared with placebo (Cook et al., 2015; Howatson et al., 2022), and one showed a significant decrease compared to placebo (Willems et al., 2015). However, one of these had a unique finding in that lactate levels increased significantly during the steady-state (SS) phase of exercise, but showed no significant difference during a 15 km time trial (Morgan et al., 2019). In the latter study, blood lactate concentrations at both the first and second lactate thresholds w showed no significant difference than before supplementation (Roberts et al., 2023). Another marker related to lactate, blood glucose, was only assessed in one study and showed no significant difference compared to the placebo group (Cook et al., 2015). Energy substrates and their oxidative metabolism related to aerobic endurance performance were also assessed. Four trials assessed fat and their oxidation, while five trials assessed carbohydrates and their oxidation. Of the four trials that measured fat-related indices, two trials showed a significant increase in fat oxidation compared with the placebo group (Cook et al., 2015; Roberts et al., 2015), one trial showed no significant change compared with placebo (de Lima Tavares Toscano et al., 2020), and another trial showed no significant difference compared with before supplementation (Roberts et al., 2023). However, in one study, despite a significant increase in fat oxidation compared with the placebo group, there was no significant difference in TFA concentration (Roberts et al., 2015). In another study, fat oxidation during high-intensity aerobic exercise did not show a significant change compared to pre-supplementation, and similarly, carbohydrates and proteins did not change significantly compared to pre-supplementation, resulting in no significant difference in heat-generated (Roberts et al., 2023). With regard to carbohydrates, one study showed a significant decrease in carbohydrate oxidation compared to placebo (Roberts et al., 2015), whereas the other four studies showed no significant differences (Cook et al., 2015; Martinez-Noguera et al., 2019; de Lima Tavares Toscano et al., 2020; Roberts et al., 2023). Only one trial evaluated TOIs and one trial evaluated FMD. However, in this study, baseline TOI was significantly increased compared to placebo, but there was no significant difference during steady-state (SS) exercise or the 15-km time trial (Morgan et al., 2019). However, there was a significant increase in FMD compared to the control group (Nho and Kim, 2022).

#### 3.5.3 Antioxidant parameters

Of the 11 trials, only two assessed antioxidant capacity or antioxidant markers. One study evaluated CAT, SOD, GSH, TBARS, and % GSSG/GSH, but found no significant differences compared to the placebo group (Martinez-Noguera et al., 2019). In another study, assessment of antioxidant capacity before and after supplementation showed a significant improvement in antioxidant activity, but there was no significant difference in lipid peroxidation response. This study also assessed TAC and MDA levels in relation to antioxidant capacity but found no significant differences compared with placebo (de Lima Tavares Toscano et al., 2020).

## 4 Discussion

The systematic review of 11 studies in this article demonstrates notable enhancements in several indicators of aerobic endurance exercise performance through polyphenol supplementation. These improvements encompass mean speed, power output, and distance covered. Notably, the most discernible enhancements were witnessed in the time taken to complete the exercise test and the time to fatigue, both of which exhibited substantial improvements when compared to the placebo group or the pre-supplementation status. Furthermore, there was a marked increase in fat oxidation. However, the results from these 11 trials also suggest limited effects of polyphenol supplements on cardiovascular function, pulmonary ventilation, gas exchange, and carbohydrate oxidation. Additionally, one of the two trials assessing antioxidant status demonstrated a significant improvement with polyphenol supplementation (de Lima Tavares Toscano et al., 2020).

This review is consistent with the findings of previous literature that polyphenol supplementation can improve aerobic endurance performance (Braakhuis and Hopkins, 2015; Somerville et al., 2017). Previously, there have been some systematic reviews and meta-analyses of the effects of polyphenols on exercise performance. However, they have certain limitations: some of the literature they included did not specify the polyphenol content of the intervention, and there is currently no review that categorizes the exercise protocols studied. Different forms of exercise have very different emphases. Aerobic endurance is crucial for endurance sports, whereas anaerobic explosive exercise emphasizes anaerobic explosiveness and strength-related metrics. This review focuses primarily on the effects of polyphenols on aerobic endurance performance in endurance sports.

This systematic review encompassed both professional athletes and sports enthusiasts (non-athletes). The focus among professional athletes was primarily on endurance sports, including marathon runners, cyclists, and triathletes. Additionally, athletes from sports such as basketball and taekwondo, which demand significant aerobic endurance during their activities, were included. Prolonged or high-intensity exercise can trigger oxidative and inflammatory responses in the body (He et al., 2016; Metsios et al., 2020; Powers et al., 2020). Sports like marathons, triathlons, and cycling time trials typically entail extended periods of competition. Conversely, basketball, characterized by rapid transitions and tactical demands, necessitates players to sustain prolonged periods of movement on the court. Thus, aerobic endurance holds particular significance in these sports. However, these prolonged and high-intensity athletic activities can induce oxidative damage to the body, ultimately impacting athletic performance. This raises the question of whether supplementation with polyphenols, a common exogenous antioxidant, can enhance the aerobic endurance performance of athletes or amateur sports enthusiasts.

### 4.1 Benefits and mechanisms of polyphenols on aerobic performance

Blackcurrants are a fruit rich in polyphenolic compounds that we use every day. In addition to small amounts of flavanols and flavonols, they also contain anthocyanins and various glycosides (Cook et al., 2015). Cyanidin is a type of flavonoid with certain anti-inflammatory (Zhu et al., 2013) and antioxidant (De la Cruz et al., 2013) properties. Besides blackcurrants, several food sources are rich in cyanidin, main fruits such as grapes, Montmorency cherries, and others (Bowtell and Kelly, 2019). Several trials included in this review used cyanidin-rich polyphenolic compounds as interventions. These included blackcurrant extract capsules (Cook et al., 2015; Willems et al., 2015), Montmorency cherry capsules (Morgan et al., 2019), Haskap berries (Howatson et al., 2022), grape and apple extract capsules (Deley et al., 2017) and grape juice (de Lima Tavares Toscano et al., 2020). Previous research consistently indicates that anthocyanins and polyphenol-rich foods have a positive impact on oxidative stress, inflammation, and muscle recovery indices (Howatson et al., 2010; Bell et al., 2014; Bell et al., 2015; Bowtell and Kelly, 2019). However, in previous reviews or meta-analyses, there has been a relative scarcity of data or analysis concerning exercise performance (Kimble et al., 2023). Looking at the combined literature, we find that supplementation with these cyanidin-rich polyphenols has shown improvements in several exercise performance-related indicators. In de Lima et al. (de Lima Tavares Toscano et al., 2020), athletes ran for an average of 59.2 ± 27.8 min and covered an average distance of 12.6 ± 6.3 km after consuming a placebo drink until exhaustion. However, after ingesting purple grape juice, they exhibited significantly better performance, running on average 9.2 min longer than the placebo group, representing an 18.7% improvement, and covering an additional 1.9 km compared to the placebo group. Similarly, in Nho et al. (Nho and Kim, 2022), the time to exhaustion during exercise was 128.9 ± 53.0 s in the placebo group and significantly increased to 134.4 ± 58.4 s in the green tea extract group. Gaelle Deley et al. found a noteworthy increase in exercise-to-fatigue time (+9.7% ± 6.0%) in the Vinitrox™ group compared to the placebo group in the endurance test (Deley et al., 2017). In Morgan et al. (Morgan et al., 2019), a randomized controlled trial showed that time trial (TT) completion time based on Montmorency cherries (1,506 ± 86 s) was 4.6% ± 2.9% faster than that based on the placebo (PL) (1,580 ± 102 s), indicating that polyphenol supplementation could reduce race completion time.

Additionally, Cook et al. (Cook et al., 2015) found that supplementing with New Zealand blackcurrant, compared with the placebo group, reduced the 16.1 km running time from 1722 ± 131 s in the control group to 1,678 ± 108 s in the supplement group, resulting in a 2.4% performance improvement. Furthermore, Howatson et al. (Howatson et al., 2022) found that the Haskap group, supplemented with Haskap berries, improved their 5 KM time trial performance by approximately 21 s compared to the placebo group, equivalent to an increase in average running speed of 0.25 km/h, representing a performance improvement of more than 2%. In 2015, Roberts et al. (Roberts et al., 2015) recruited 14 men to participate in a randomized controlled trial by randomly assigning either a supplement capsule containing dGTE or a placebo for 4 weeks, followed by a 40-min performance trial at weeks 0, 2, and 4. The use of dGTE led to a gradual increase in distance covered, from 20.23 ± 0.54 km in week 0–21.77 ± 0.49 km in week 2, and finally, to 22.43 ± 0.40 km in week 4, showing a significant increase of 10.9%. Similarly, a significant increase in average power output was observed. The average power output increased by 17.9% from week 0 (162.06 ± 10.08W) to week 2 (191.08 ± 10.85W), and from week 0 to week 4 (198.91 ± 8.61W) increased by 22.7%. This suggests that supplementation with polyphenols, primarily cyanidin-rich compounds, benefits both recreational and professional athletes in terms of aerobic endurance performance, despite variations in supplement form and dosage. The mechanism behind the improvement in exercise performance with cyanidin supplementation is related to the improvement in endothelial function. The mechanism by which supplementing anthocyanins improves exercise performance is related to the enhancement of endothelial function. NO is an efficient vasodilator capable of mediating and regulating vascular function and blood flow during exercise (Ferguson et al., 2013; Lee et al., 2015). By increasing the production of endothelium-derived vasodilator NO, supplementation of anthocyanins can mediate endothelium-dependent vasodilation induced in the thoracic aorta of rats (Nakamura et al., 2002). Another non-glycosylated anthocyanin in blackcurrants, delphinidin, can also increase endothelial NO production to vasodilate blood vessels by elevating intracellular Ca^2+^ concentration in endothelial cells. This increase in NO, along with a reduction in NO free radical breakdown, contributes to enhanced peripheral blood flow (Martin et al., 2010).

Recruiting 12 elite athletes, Nho et al. (Nho and Kim, 2022) conducted a study to compare the effects of GSE and placebo on endothelial function during 14 days of progressive cycling. Brachial endothelial function was assessed using Flow-Mediated Dilation (FMD). The results indicated that GSE led to an increase in brachial artery diameter induced by FMD (14.4% ± 5.2% vs. 17.6% ± 4.5%). The study demonstrated that long-term supplementation of GSE improved endurance performance, possibly attributed to the vasodilation of active skeletal muscle mediated by enhanced endothelial functio. (Howatson et al., 2022). Haskap berries containing cyanidin-3-O-glucoside (C3G) have been shown to increase mitochondrial biogenesis, improve muscle function, and enhance exercise performance in rodents (Matsukawa et al., 2017; Saclier et al., 2020). In the literature we examined, a study on Haskap berries supplementation caught our attention. The research involved 30 male recreational runners in a double-blind, placebo-controlled, independent group design. The participants were randomly assigned to either the Haskap berries intervention or an isocaloric placebo control to investigate the impact of Haskap berries on parameters related to endurance running performance. The study observed slight changes in heart rate and VO_2max_ at submaximum intensity. Notably, during the VO_2peak_ test, the Haskap group showed a 20-s extension in exercise-to-fatigue time, representing a meaningful improvement in the context of human running performance (Deley et al., 2017). During intense exercise, upregulation of antioxidant genes and protein expression mediated by Nrf2 through C3G helps maintain muscle function. Therefore, Haskap berries may potentially enhance performance by modulating endothelial function pathways through C3G mediation (Xu et al., 2004; Edwards et al., 2015). It is therefore speculated that the improvement in vascular endothelial function leads to increased O_2_ utilization at low intensities. This may explain changes in associated fatigue times and TT race performance. Future research should refine this concept more systematically and strive to comprehensively investigate various aspects of the proposed mechanism to gain a more enriched understanding of the impact of dietary anthocyanins on exercise performance. Attempting to elucidate a singular mechanism through simplified approaches may not be practical.

### 4.2 Effects of polyphenols on energy metabolism during aerobic exercise

In this review, we found that consuming polyphenols may have an impact on fat metabolism. In Cook et al. (Cook et al., 2015) well-trained endurance athletes who supplemented with blackcurrant extract capsules for 1 week showed a significant difference in the outcome measure FATox between the experimental group (0. 44 ± 0. 12) and the placebo group (0. 37 ± 0. 15). This suggests that supplementation with blackcurrant extract capsules may enhance whole-body fat oxidation during moderate-intensity exercise. The appearance of lactate may have been influenced by the effect of anthocyanins on substrate oxidation, potentially leading to an increased contribution of fat oxidation at relatively low intensities (Willems et al., 2015). Tsuda et al. (Tsuda et al., 2005) in 2005 found that glycoside-treated adipocytes upregulated genes related to fat metabolism and signaling. Similarly, Benn et al. (Benn et al., 2014) found that long-term intake of blackcurrant extract increased energy metabolism-related genes and mRNA levels in C57BL/6J mice. Therefore, the increase in fat oxidation may result from the combined effects of multiple pathways including the upregulation of genes related to fat oxidation, transportation of fatty acids to mitochondria, enhanced availability of nitric oxide, and increased peripheral blood flow. Similar findings were replicated in another study involving green tea extract. Roberts et al. (Roberts et al., 2015) enrolled fourteen recreationally active males and randomly administered either green tea extract or a placebo. The results revealed a noteworthy increase in the total fat oxidation rate within the dGTE group, rising from 0.241 ± 0.025 g/min to 0.301 ± 0.009 g/min, marking a substantial increase of 24.9%. The benefits of green tea extract on the human body are primarily associated with the catechin polyphenols, a significant proportion of which is EGCG (Randell et al., 2014), EGCG upregulates cell signaling not only through antioxidant protective mechanisms but also via the mediation of PGC1α (Hodgson et al., 2013), SIRT1 and the mAPK pathway (Vincent et al., 2013; Kim et al., 2014). Over a longer period (>4 weeks), moderate doses of EGCG may promote the upregulation of genes associated with fat metabolism during exercise, thereby enhancing whole-body fat oxidation. Some studies also suggest that EGCG may improve glucose tolerance, insulin sensitivity, and adiponectin levels, further supporting this notion (Potenza et al., 2007; Venables et al., 2008). In contrast, Martinez et al. (Martinez-Noguera et al., 2019) in 2019 showed that acute supplementation with hesperidin increased activation of the intracellular signaling pathway AMPK, induced changes in PGC1α activity, and promoted the use of fat as an energy substrate. Increased FATox implies a degree of glycogen sparing during moderate-intensity exercise. Howatson et al. (Howatson et al., 2022) found that HR and VO_2_ were lower at a certain exercise intensity (lactate threshold). As fat oxidation consumes more oxygen than carbohydrate oxidation, fat may not be the preferred energy substrate under these circumstances.

Similarly, at moderate intensity (50% VO_2max_), subjects’ FATox increased from 0. 241 ± 0. 025 to 0. 301 ± 0. 009 g-min-1 after supplementation with green tea extract, an improvement of approximately 24. 9%. However, the magnitude of the increase was more significant than in the previous study. The subjects in this study were recreationally active, so it has been suggested that the combined effect of exercise training and polyphenol supplementation may be more appropriate for untrained individuals (Randell et al., 2014). In the studies considered, grape seed extract exhibited no significant impact on the performance of primary basketball players (Nho and Kim, 2022). However, among non-endurance sports athletes, supplementation with carob demonstrated a significant improvement in maximal aerobic velocity (MAV) and performance in the yoyo test compared to baseline for professional taekwondo athletes. Gaamouri et al. (Gaamouri et al., 2019) recruited 23 taekwondo athletes for their experiment. Prior to the study intervention, there were no significant differences between the groups. After the intervention, the carob supplement group showed significant improvements of 92.43% in distance and 12.18% in MAV, whereas the placebo group only exhibited improvements of 40.37% and 4.95%, respectively. This result indicates that polyphenol supplementation can effectively enhance the athletic performance of professional athletes. Carbohydrates and fats are the most important substrates for energy metabolism during exercise. However, the proportion of energy contribution from these substrates varies with different exercise durations and intensities. During low to moderate-intensity exercise (up to 60% of VO_2max_), the absolute value of fat oxidation increases (Dandanell et al., 2017). As exercise intensity further increases, the absolute rate of fat oxidation decreases, and carbohydrates become the primary energy substrate (Dandanell et al., 2017). In the process of exercise, the ability to oxidize fats at a high rate is considered an advantage for endurance-trained athletes. Muscle glycogen stores are relatively small; therefore, theoretically, any intervention that enhances skeletal muscle fat oxidation capacity could lead to glycogen sparing and thereby enhance endurance (Randell et al., 2014). Endurance exercise training leads to skeletal muscle adaptations that favor fat metabolism (Tunstall et al., 2002). Trained individuals exhibit higher absolute fat oxidation rates compared to untrained populations (Nordby et al., 2010). However, the increase in fat oxidation following polyphenol intervention is relatively smaller in trained individuals compared to untrained ones, explaining the more significant impact of polyphenols on exercise performance in untrained individuals or those engaged in non-endurance activities. In recent years, the topic of olive-derived supplements has gained popularity. In Roberts et al. (Roberts et al., 2023)in 2023, an olive-derived supplement rich in hydroxytyrosol, an important polyphenol, may support endogenous antioxidant mechanisms related to mitochondrial respiratory capacity, such as upregulation of PGC-1α (Feng et al., 2011; Wood Dos Santos et al., 2018), This study showed no difference in VO_2max_ performance during 75% moderate-intensity exercise, which may be related to regular aerobic exercise habits. Further research is needed to refine the types of polyphenols, dosages, and exercise protocols for different populations.

### 4.3 Supplementation time and supplemental dose

This review suggests that prolonged use of specific polyphenol supplements may promote whole-body fat oxidation during moderate-intensity exercise, which may benefit aerobic endurance performance in both professional athletes and sports enthusiasts. This may have implications for how people utilize energy substrates and plan their dietary strategies. In terms of supplementation strategies, only three out of the 11 trials included acute supplementation before exercise. Acute polyphenol supplementation was found to improve exercise performance compared to continuous supplementation, although the effect was not statistically significant. In Martinez et al. (Martinez-Noguera et al., 2019), acute ingestion of 500 mg of 2S-hesperidin (Cardiose^®^) was administered to investigate its impact on athletic performance. The best data from repeated sprint trials showed significant differences between Cardiose^®^ and placebo in mean power (+2.27%), maximum speed (+3.23%), and total energy (+2.64%). While acute polyphenol supplementation improved performance in repeated sprint tests, the average performance did not differ significantly from the placebo group. Similarly, in a separate study, Deley et al. (Deley et al., 2017) explored the effects of acute intake of grape and apple polyphenols on endurance exercise capacity. Volunteers were randomly assigned to either take 500 mg of polyphenols or a placebo the night before and 1 h before the test. The mean duration of the maximum endurance test significantly increased in the polyphenol group compared to the placebo group (+9.7% ± 6.0%). The maximal perceived exertion was reached later with polyphenols (+12.8% ± 6.8%). Another study (de Lima Tavares Toscano et al., 2020) investigated the effects of a single dose of grape juice on runners’ physical performance. In a running test up to exhaustion (80% VO_2peak_) after consuming a placebo drink, athletes ran for an average of 59.2 ± 27.8 min and covered an average of 12.6 ± 6.3 km. During the course after ingestion of purple grape juice, they demonstrated significantly enhanced performance, running for an average of 9.2 min longer, representing an 18.7% improvement, and a 1.9-km increase in distance compared to the placebo group. Among the studies on acute supplementation included, this one exhibited the most substantial improvement in athletic performance. However, in terms of endurance exercise distance, long-term polyphenol intake yielded a greater enhancement in athletic performance. Gaamouri et al. (Gaamouri et al., 2019) designed a 6-week double-blind randomized parallel fully controlled training study with pre- and post-measurements. Analysis of aerobic activity for 6 weeks before and after carob supplementation showed that the total distance covered increased from 847.2 ± 473.9 m to 1,494.9 ± 619.2 m, representing an increase of 92.43%. Regarding average power output, there was no significant change in average power output in Martinez et al. (Martinez-Noguera et al., 2019) results which is acute polyphenols supplementation. On the other hand, Roberts et al. (Roberts et al., 2015) found in his 2015 study that supplementation of green tea extract capsules for 4 weeks resulted in a notable increase in average power output with dGTE by 17.9%, or 29.02 ± 5.53W from week 0 (162.06 ± 10.08W) to week 2 (191.08 ± 10.85W), and by 22.7%, or 36.85 ± 3.20W from week 0 to week 4 (198.91 ± 8.61W). Metabolic parameters also showed no significant differences in these two trials. This may be due to the small sample size, as only two articles existed. Because the bioavailability of phenolic compounds in different supplements has not been well established, the availability of supplements cannot be determined before starting an exercise program. Caution should be taken when comparing studies involving different types of polyphenols, as their bioavailability may vary and subsequent interactions with other nutrients taken at the same time may influence the results of the comparison (Myburgh, 2014).

In this review, the average intake of polyphenols was 229. 04 mg/day, which is equivalent to about 66 g of dark chocolate, 83 mg of green tea, and 99 mg of mixed berries (blackcurrants, strawberries, and blackberries). In these 11 trials, specific individuals and combinations of polyphenols were used as interventions, and the forms of intake varied. Five trials used capsules (Cook et al., 2015; Roberts et al., 2015; Deley et al., 2017; Morgan et al., 2019; Nho and Kim, 2022), three trials used water-soluble forms (Gaamouri et al., 2019) or yoghurt (Howatson et al., 2022) as interventions, and there was also oral consumption through snacks or canned forms (de Lima Tavares Toscano et al., 2020; Roberts et al., 2023). Of these, only one study supplemented carob pods in a water-soluble form, and the participants in this study increased their endurance running distance by 92. 43%, which was a significantly greater improvement compared to other supplementation methods (Gaamouri et al., 2019). However, this supplementation method was only mentioned in one study in the literature, making it difficult to draw qualitative conclusions. Further research is needed to address the complex issue of the relative effects of different forms of polyphenol intake on athletic performance. Analysis of the performance indicators in the 11 studies showed that long-term polyphenol supplementation can significantly improve endurance exercise performance. The most significant indicator was a 92. 43% increase in endurance running distance and this trial had the longest duration of polyphenol supplementation of all 11 trials, lasting 6 weeks (Gaamouri et al., 2019). In contrast, another study found that acute polyphenol supplementation resulted in an increase in endurance running distance of approximately 18. 7% (de Lima Tavares Toscano et al., 2020). This suggests that long-term polyphenol supplementation may have a more pronounced effect than acute supplementation.

It is important to note that existing research suggests that high doses of antioxidant supplementation appear to have a detrimental effect on endurance performance (Paulsen et al., 2014). High doses of antioxidant supplements can shut down those cell signaling pathways sensitive to redox changes, thereby reducing the synthesis of new muscle mitochondria and the production of endogenous antioxidants (Kang et al., 2009; Hawley et al., 2011; Feng et al., 2013). Crucially, the health benefits and performance enhancements brought about by endurance training seem to be somewhat related to this cellular adaptation (Coffey and Hawley, 2007; Ristow and Zarse, 2010). The results showed that supplementation with high doses of antioxidants reduced the increase in cytochrome c oxidase subunit IV(COX4) induced by endurance training in the vastus lateralis muscle (Paulsen et al., 2014). While antioxidant supplementation does not affect the short-term improvement in endurance performance, it may negate the beneficial long-term adaptive effects of endurance training on cells. This suggests that individuals who frequently engage in endurance training should be cautious about using high doses of antioxidants. In contrast, in this study, long-term supplementation with polyphenols had more pronounced effects than acute or short-term ones. This may be related to the dose of polyphenols included in the 11 studies and the different bioavailability of polyphenols compared to other antioxidants (Di Lorenzo et al., 2021), necessitating future research to explore whether high-dose polyphenol supplementation affects those signaling pathways related to improvements in endurance performance.

### 4.4 The potential of polyphenols

It should be noted that due to their complex structures and higher molecular weights, polyphenols are not completely absorbed in the gastrointestinal tract. Instead, they are primarily biotransformed into low molecular weight, biologically active phenolic metabolites in the large intestine through the action of the gut microbiota (Hill et al., 2014; Ma and Chen, 2020). These low molecular weight metabolites produce antimicrobial substances, regulate the host immune system, and inhibit the production of bacterial toxins. They also have a certain preventive effect on some chronic diseases (Cerdá et al., 2005; Espín et al., 2013; Markowiak and Śliżewska, 2017). Furthermore, polyphenols can modify the composition of the gut microbiota, promoting the growth of beneficial microbes, such as lactobacilli and bifidobacteria, which are two major probiotics beneficial for human health (Wang et al., 2022). In interaction, the gut microbiota also metabolizes polyphenols, producing biologically active metabolites, such as short-chain fatty acids, which further impact the host’s health (Wang et al., 2022). The bioactivity of polyphenols and their metabolites in the body is likely mediated through these metabolites. These metabolites are generated in the body, and recent studies have confirmed that these molecules may have antioxidant and anti-inflammatory properties (Di Lorenzo et al., 2021). The bioavailability of polyphenols in the small intestine is low, mainly due to their interactions with food matrices, liver-mediated metabolic processes (both primary and secondary metabolism), and metabolic processes in the gut and microbiota (Di Lorenzo et al., 2021). However, the activity demonstrated by these compounds through their metabolites in the organism suggests that long-term intake of polyphenols can stabilize and alter the composition of the gut microbiota, thus promoting more beneficial health effects. The aforementioned supplementation with polyphenols can improve the utilization rate of fats as an energy substrate, and polyphenols are also positively correlated with anti-lipogenesis. Polyphenols have been shown to effectively activate the browning of adipose tissue, reducing obesity and lipid accumulation by inducing the browning of beige fat cells (Hu et al., 2020). Daily intake of beverages rich in catechins can increase the density of brown adipose tissue in healthy young women, supporting polyphenols’ role in brown fat genesis (Nirengi et al., 2016). In mice on a high-energy diet, ferulic acid accelerated thermogenesis and mitochondrial synthesis in brown adipose tissue and inguinal white adipose tissue (Han et al., 2018). Brown adipose tissue consumes energy more efficiently, contributing to weight management and improved body fat distribution, which is very important for maintaining good athletic performance and endurance. In recent years, polyphenols have attracted attention for their neuroprotective effects, showing potential effectiveness in reversing neurodegenerative pathology and age-related cognitive decline. Animal studies have shown that blueberries can improve spatial memory deficits in rats, (−)-epigallocatechin enhanced the retention of spatial memory in mice, curcumin could break down plaques and restore neurites in Alzheimer’s disease models, and resveratrol reduced Aβ aggregation in rat hippocampal cells by activating specific protein kinases (Casadesus et al., 2004; Dasgupta and Milbrandt, 2007; Garcia-Alloza et al., 2007; Praag et al., 2007). However, the direct link between polyphenols and improvements in neural health has not been clearly established.

### 4.5 Limitations

This review has some limitations that should be considered. Firstly, a meta-analysis could not be performed due to the heterogeneity in study design, populations, training status/types, and supplementation methods. Secondly, some studies used VO_2max_ as a recognized performance indicator, but some subjects may have reached VO_2peak_ during testing due to their training habits and routines, leading to inherent measurement errors that could potentially affect the data (McArdle et al., 2006). Additionally, we did not classify the participants, which to some extent affected the comparison of the impact of polyphenols on exercise performance. Each of the 11 articles included in this review had varying durations of intervention and small sample sizes, making it difficult to effectively conduct a qualitative analysis of polyphenol supplementation. In addition, in the articles by Martínez et al. (Martinez-Noguera et al., 2019) and Willems et al. (Willems et al., 2015) the participants were aware of the allocation scheme or were made aware of it, which may have biased the results. Furthermore, in Willems’s article (Willems et al., 2015), there was a lack of complete reporting of the performance scores of each participating subject, which could also introduce bias in the results. There are several important considerations for future research. Firstly, more emphasis should be placed on investigating the bioavailability of polyphenols, as this may help to determine the optimal dosage and supplementation methods for different types of exercise, and thus have beneficial effects on athletic performance. Secondly, considering participant categorization and specialization is important. Further investigations can ascertain the potential applications of polyphenols and their energetic effects on various categories of participants and specialized athletes. In addition, few studies have included comprehensive dietary controls. As the sources of polyphenol intake are diverse, efforts should be made to quantify the intake of polyphenols in participants’ diets using placebo-controlled designs, as individuals with low intakes may show more positive responses to dietary interventions. This will help to better explain the research findings.

## 5 Conclusion

In summary, the results of the 11 studies indicate that flavonoid-rich compounds, providing a total of 208 mg of polyphenols and 14.4 mg of flavonoids per 40 g in a water-soluble form, demonstrated the most noticeable improvement in exercise performance during a 6-week supplementation period (Gaamouri et al., 2019). Supplementation with polyphenols or polyphenol complexes may improve aerobic endurance performance and promote fat oxidation in the human body. However, there is not enough research to confirm the effects of polyphenols or polyphenol complexes on other outcomes (cardiovascular, antioxidant, gender) to draw definitive conclusions. Further research is needed to clarify the potential benefits of polyphenols or polyphenol complexes on other indicators (dosage, timing, controversies).

## Data Availability

The original contributions presented in the study are included in the article/Supplementary material, further inquiries can be directed to the corresponding author.
